# Robotic repair of an esophago–left main bronchial fistula using a pedicled autologous pericardial patch and latissimus dorsi muscle flap: a novel technique after chemoradiotherapy for cT4b esophageal cancer

**DOI:** 10.1007/s10388-026-01235-x

**Published:** 2026-08-28

**Authors:** Tomoki Makino, Shigeto Nakai, Tomohira Takeoka, Kotaro Yamashita, Kazuya Kuroda, Koji Tanaka, Takashi Kanou, Tateki Kubo, Yasushi Shintani, Shigeru Miyagawa, Hidetoshi Eguchi, Yuichiro Doki

**Affiliations:** 1 Department of Gastroenterological Surgery, The University of Osaka Graduate School of Medicine, 2-2-E2, Yamada-Oka, Suita, Osaka 565-0871 Japan; 2 Department of Gastroenterological Surgery, Sakai City Medical Center, Osaka, Japan; 3 Department of Plastic Surgery, The University of Osaka Graduate School of Medicine, Suita, Osaka Japan; 4 Department of General Thoracic Surgery, The University of Osaka Graduate School of Medicine, Suita, Osaka Japan; 5 Department of Cardiovascular Surgery, The University of Osaka Graduate School of Medicine, Suita, Osaka Japan; 6 Department of Gastroenterological Surgery, Osaka General Medical Center, Osaka, Japan

**Keywords:** Robotic surgery, Airway fistula, Pedicled pericardial patch, Esophageal cancer, cT4b

## Abstract

Once an esophago–airway fistula develops during chemoradiotherapy for cT4b esophageal cancer, further curative treatment is often precluded. We describe a robotic technique for closure of a large esophago–left main bronchial fistula using a pedicled autologous pericardial patch reinforced with a latissimus dorsi muscle flap. A 66-year-old woman with squamous cell carcinoma of the middle to upper thoracic esophagus invading the left main bronchus developed a fistula after definitive chemoradiotherapy. After temporary airway stenting, salvage surgery was performed in the semi-prone position under single-lung ventilation and CO₂ pneumothorax. Following thoracic esophagectomy, a horseshoe-shaped pedicled pericardial patch was created robotically, flipped cranially, and sutured to the bronchial defect. A right latissimus dorsi muscle flap was then introduced into the right thoracic cavity through a second intercostal opening and robotically fixed over the repair. To our knowledge, this is the first report of this robotic technique.

**Supplementary Information:**

The online version contains supplementary material available at https://doi.org/10.1007/s10388-026-01235-x.

## Introduction

Airway fistula after chemoradiotherapy for esophageal cancer is a rare but life-threatening condition. Large fistulas around the carina or main bronchus are particularly difficult to manage because the surrounding tissues are often inflamed, ischemic, infected, or severely fibrotic. Although endoscopic or airway stenting may be useful as temporary stabilization, durable closure of a large fistula generally requires removal of the contaminated esophageal source, secure airway repair, and interposition of well-vascularized tissue. Several vascularized flaps have been used for tracheoesophageal or bronchoesophageal fistulas. Pericardial flaps are attractive because they are adjacent to the tracheobronchial tree, relatively noncompliant, and can separate the airway from the alimentary tract. Wheatley and Coran reported the usefulness of vascularized pericardial interposition for recurrent tracheoesophageal fistula, emphasizing that the flap isolates the tracheal and esophageal suture lines [1]. Yasuda et al. later described open repair of a post-chemoradiotherapy tracheoesophageal fistula using a pedicled posterior pericardial flap reinforced with a latissimus dorsi muscle flap [2]. However, to our knowledge, robotic creation and suturing of a pedicled autologous pericardial patch for esophago–bronchial fistula after chemoradiotherapy for esophageal cancer has not been reported. We describe a robotic technique for repair of a large esophago–left main bronchial fistula using a pedicled autologous pericardial patch combined with a latissimus dorsi muscle flap.

### Case presentation and indication

**Fig. 1:**
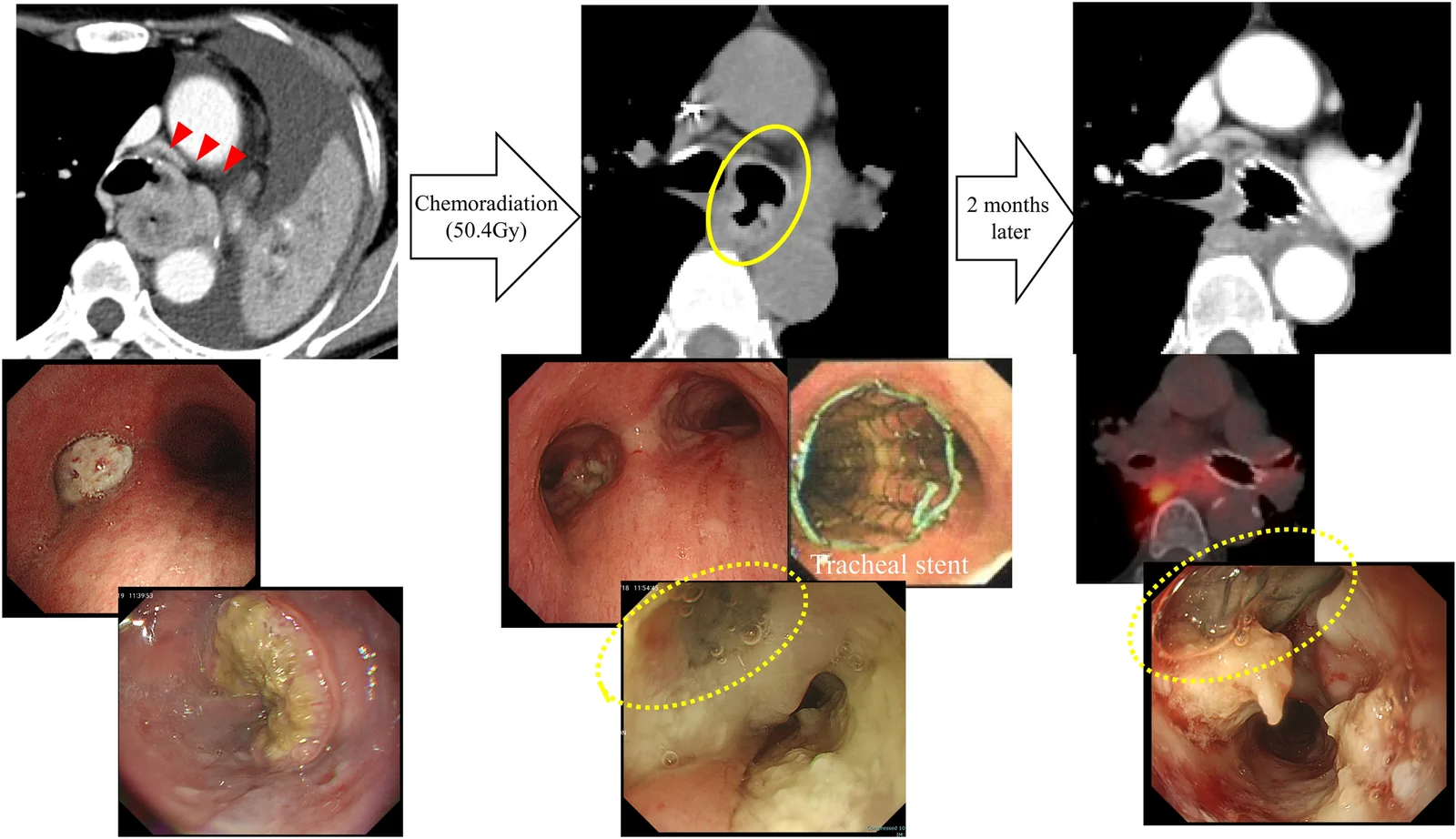
Preoperative imaging findings before and after chemoradiotherapy. Left: Pretreatment imaging demonstrated thoracic esophageal cancer invading the left main bronchus, with obstruction at the level of the left main bronchus and resultant collapse of the left lung. Middle: An esophago–left main bronchial fistula developed during chemoradiotherapy; the fistula is indicated by the yellow solid line, and an airway stent was placed. Right: Imaging obtained two months after completion of chemoradiotherapy showed persistence of the fistula. In the endoscopic images in the bottom row of the middle and right panels, the airway stent is visible through the fistula and is indicated by the yellow dashed line

A 66-year-old woman was diagnosed with squamous cell carcinoma of the middle to upper thoracic esophagus invading the left main bronchus, classified as cT4bN2M0, stage IVA (Fig. 1). The tumor caused complete obstruction of the left main bronchus with collapse of the left lung. She received definitive chemoradiotherapy with cisplatin plus 5-fluorouracil and 50.4 Gy irradiation. Tumor regression was followed by formation of a fistula between the esophagus and the left main bronchus (Fig. 1). Oral intake was discontinued, and an Ultraflex tracheobronchial covered self-expanding metallic stent, with an expanded outer diameter of 14 mm and an expanded length of 60 mm, had been placed at the referring hospital. The fistula was located approximately 10 mm distal to the tracheal bifurcation. The stent was positioned to cover the fistula, with its proximal edge protruding approximately 5 mm into the trachea and its distal edge located approximately 40 mm distal to the fistula, proximal to the secondary carina. She was referred to our institution for definitive surgical treatment. Preoperative PET-CT showed no distant metastasis and only faint uptake at the primary site (Fig. 1). Because the fistula was large and located from the carina to the proximal left main bronchus, and because the esophagus was the contaminated source, we planned thoracic esophagectomy followed by robotic airway repair with a pedicled autologous pericardial patch and vascularized muscle reinforcement.

### Operative technique

**Fig. 2:**
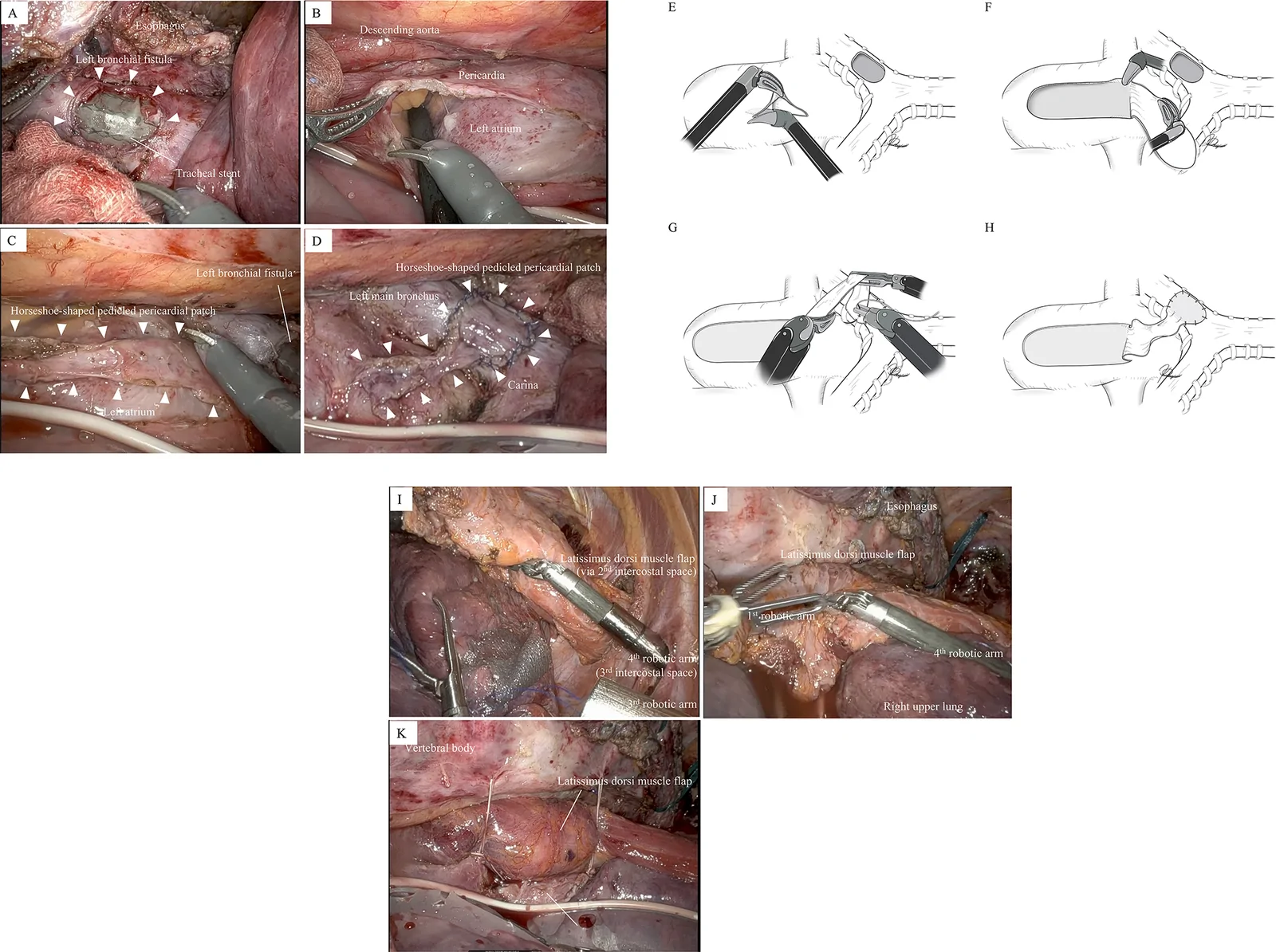
Intraoperative findings and schema of robotic fistula repair. **A** A large fistula was identified between the esophagus and the left main bronchus after mobilization of the thoracic esophagus. **B**, **C** A horseshoe-shaped pedicled autologous pericardial patch was designed near the tracheal bifurcation and harvested with sufficient length to reach the bronchial defect without tension. **D** The pedicled pericardial patch was flipped cranially and sutured to the bronchial defect. **E–H** Schematic illustration of robotic repair of the airway defect using a pedicled autologous pericardial patch. **I–K** A pedicled latissimus dorsi muscle flap was introduced into the right thoracic cavity through the second intercostal space, routed medially along the vertebral body to allow its tip to reach the bronchial fistula, and fixed over the repaired fistula to reinforce the pericardial patch

The patient was placed in the semi-prone position. Single-lung ventilation and CO₂ pneumothorax at 10 mmHg were used. After port placement, the thoracic esophagus was mobilized robotically. Dense scar tissue was present around the previous tumor site and the left main bronchus. Careful dissection was performed to avoid injury to the airway and adjacent major vessels. When the esophagus was detached from the airway, a large fistula measuring approximately 3 × 2 cm was exposed from the region of the carina to the proximal left main bronchus (Fig. 2A). Frozen-section assessment of the dissected margin showed no viable tumor. Thoracic esophagectomy was completed, and immediate reconstruction was deferred. A pedicled autologous pericardial patch was then created robotically (Fig. 2B,C). The posterior pericardium adjacent to the fistula was incised in a horseshoe configuration, leaving the cranial side near the tracheal bifurcation as the pedicle. The flap was designed to be wide enough to cover the bronchial defect without tension and long enough to rotate cranially into the fistula. Before suturing, we confirmed that the patch reached the bronchial defect without tension, twisting, excessive traction, or kinking of the pedicle to preserve flap perfusion. The pericardial flap was then flipped cranially and placed over the bronchial opening. The patch was fixed to the edge of the membranous airway defect with interrupted absorbable monofilament (4–0 PDS^®^) sutures. Thirteen stitches were placed circumferentially to achieve airtight closure while avoiding excessive narrowing of the left main bronchus (Fig. 2D). The key steps of pedicled pericardial patch creation and cranial flipping are shown schematically in Fig. 2E–H. After completion of the pericardial patch repair, a right latissimus dorsi muscle flap was harvested by the plastic surgery team in the same semi-prone position. The thoracodorsal vessels were preserved as the vascular pedicle, and the flap was introduced into the right thoracic cavity through a minimal second intercostal opening. It was then routed medially along the vertebral body to reach the left main bronchial defect. The minimal opening and the flap itself helped maintain CO₂ pneumothorax during robotic manipulation. Because the fourth robotic arm was placed in the third intercostal space, the transferred flap did not interfere with robotic arm movement during positioning and fixation (Fig. 2I, J). The muscle flap reinforced the pericardial patch and separated the airway repair from the contaminated esophageal bed (Fig. 2K, Video 1). A cervical esophagostomy was created, and the operation was completed without immediate reconstruction.

### Postoperative course and pathological findings

**Fig. 3:**
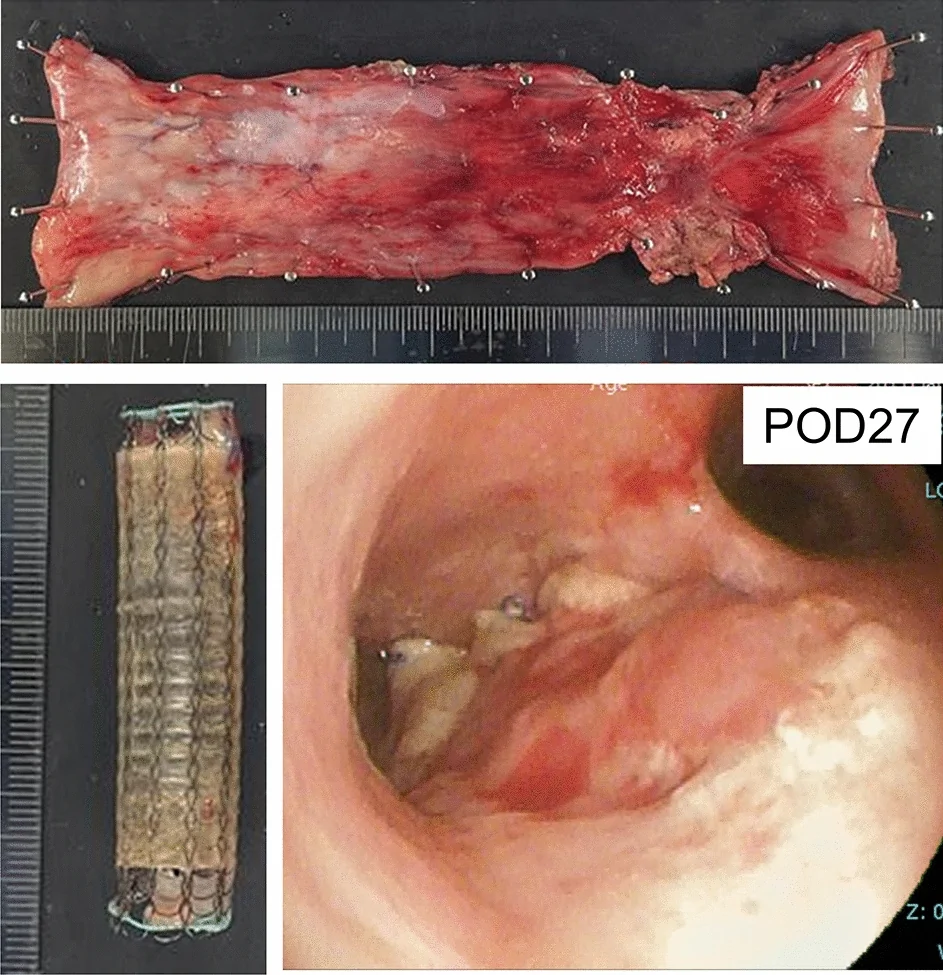
Resected specimen, removed airway stent, and postoperative bronchoscopic finding. Upper: Resected esophageal specimen showing pathological complete response after chemoradiotherapy. Lower left: Removed airway stent. Lower right: Bronchoscopic finding on postoperative day 27 after airway stent removal, showing closure of the repaired fistula without recurrent fistula

The patient developed a mild cerebral infarction related to postoperative arrhythmia, but no major respiratory complication, recurrent fistula, or patch-related complication occurred. Histopathological examination of the resected specimen demonstrated a pathological complete response, with a histological treatment effect of grade 3 (ypT0N0M0 Stage 0) (Fig. 3). The airway stent was removed three weeks after surgery without respiratory deterioration (Fig. 3). Gastric conduit reconstruction was performed three months after the initial surgery. At the 6-month follow-up, the patient remained free from fistula recurrence and tumor recurrence.

### Technical points

The first technical point is the sequence of repair. The pericardial patch should be created after the thoracic esophagus has been removed from the airway. This provides direct access to the posterior pericardium and allows the shortest route to the tracheobronchial defect. Yasuda et al. emphasized that the posterior pericardial flap is well suited for defects near the carina because it can be raised in continuity with the operative field after esophagectomy and turned toward the airway defect with minimal tension [2]. For patch fixation, we used interrupted absorbable monofilament sutures. In airway reconstruction, both absorbable and nonabsorbable sutures are commonly used depending on the surgeon’s preference and the clinical setting. Absorbable monofilament sutures were selected to provide sufficient initial fixation while potentially reducing long-term foreign body exposure in the airway. Interrupted sutures allowed precise adjustment of each stitch and suture tension in the deep, narrow field, while reducing the risk of excessive narrowing of the left main bronchus or accidental needle bites into the airway stent. The second point is the use of vascularized autologous tissue. In post-chemoradiotherapy cases, wound healing is impaired by reduced blood flow, infection, and severe scarring. A patch for a large airway defect also requires sufficient stiffness to resist intratracheal pressure and avoid endoluminal protrusion. A pedicled pericardial patch provides both vascularity and low compliance, whereas a latissimus dorsi muscle flap adds robust vascularized coverage. Previous reports have described pericardial, muscle, and thymic pedicle flaps for tracheoesophageal fistula repair [1–5]. In the present technique, the pericardium served as the structural airway patch, whereas the latissimus dorsi muscle flap was used not to provide direct blood supply to the pericardial patch itself, but as a bulky, well-vascularized autologous backing tissue over the repaired airway defect. Because the fistula was large, irradiated, contaminated, and located distally at the left main bronchus, we selected a pedicled latissimus dorsi muscle flap for sufficient tissue volume and reach. Direct evidence that the muscle flap enhances vascularization of the pericardial patch itself is limited, and this remains a limitation of the present technique. The third point is the value of the robotic platform. The defect was located near the carina and proximal left main bronchus, where conventional open/thoracoscopic suturing is technically demanding. Robotic magnified three-dimensional vision, articulated instruments, and tremor filtration enabled precise flap design and interrupted suturing in a narrow, scarred mediastinum. These features may be particularly useful when a large fistula must be repaired under pneumothorax after chemoradiotherapy. Lastly, intraoperative ventilation and airway stent management require careful planning. In this case, an airway stent within the fistula enabled stable single-lung ventilation during robotic repair. During patch fixation, we avoided accidental needle bites into the expanded stent by minimally displacing the stent only when necessary to confirm the space behind the membranous defect. Excessive displacement was avoided because it could have allowed substantial CO₂ inflow from the pneumothorax into the airway. The patch was fixed with interrupted sutures under magnified robotic vision, allowing precise needle placement while maintaining stable ventilation. Without an airway stent, fistula exposure after esophageal mobilization may cause CO₂ inflow into the airway and rapid elevation of arterial CO₂ levels. If the fistula is sufficiently distant from the secondary carina, advancing the tip and cuff of a double-lumen tube distal to the defect can help maintain ventilation; otherwise, preparation for extracorporeal respiratory support should be considered.

## Conclusion

Robotic repair with a pedicled autologous pericardial patch reinforced by a latissimus dorsi muscle flap is a feasible option for a large esophago–left main bronchial fistula after chemoradiotherapy for cT4b esophageal cancer. To our knowledge, this is the first report of this robotic technique. The method combines the structural strength and proximity of the pericardium with the vascularized coverage of a muscle flap, while taking advantage of robotic precision in a severely scarred mediastinum.

## Supplementary Information

Below is the link to the electronic supplementary material.

Supplementary file1 (MPG 80802 KB) Video 1 Robotic repair of an esophago–left main bronchial fistula using a pedicled autologous pericardial patch and latissimus dorsi muscle flap after chemoradiotherapy for cT4b esophageal cancer

## Data Availability

The data underlying this article are not publicly available due to patient privacy concerns. Further inquiries may be directed to the corresponding author.
