# Correction: Robotic repair of an esophago–left main bronchial fistula using a pedicled autologous pericardial patch and latissimus dorsi muscle flap: a novel technique after chemoradiotherapy for cT4b esophageal cancer

**DOI:** 10.1007/s10388-026-01258-4

**Published:** 2026-10-13

**Authors:** Tomoki Makino, Shigeto Nakai, Tomohira Takeoka, Kotaro Yamashita, Kazuya Kuroda, Koji Tanaka, Takashi Kanou, Tateki Kubo, Yasushi Shintani, Shigeru Miyagawa, Hidetoshi Eguchi, Yuichiro Doki

**Affiliations:** 1 Department of Gastroenterological Surgery, The University of Osaka Graduate School of Medicine, 2-2-E2, Yamada-Oka, Suita, Osaka 565-0871 Japan; 2 Department of Gastroenterological Surgery, Sakai City Medical Center, Osaka, Japan; 3 Department of Plastic Surgery, The University of Osaka Graduate School of Medicine, Suita, Osaka Japan; 4 Department of General Thoracic Surgery, The University of Osaka Graduate School of Medicine, Suita, Osaka Japan; 5 Department of Cardiovascular Surgery, The University of Osaka Graduate School of Medicine, Suita, Osaka Japan; 6 Department of Gastroenterological Surgery, Osaka General Medical Center, Osaka, Japan

**Correction to: Esophagus**

**https://doi.org/10.1007/s10388-026-01235-x**

Panels from E to K of Fig. 2 were missing from this article. For completeness and transparency, the complete version of Fig. 2 is displayed below.

**Fig. 2:**
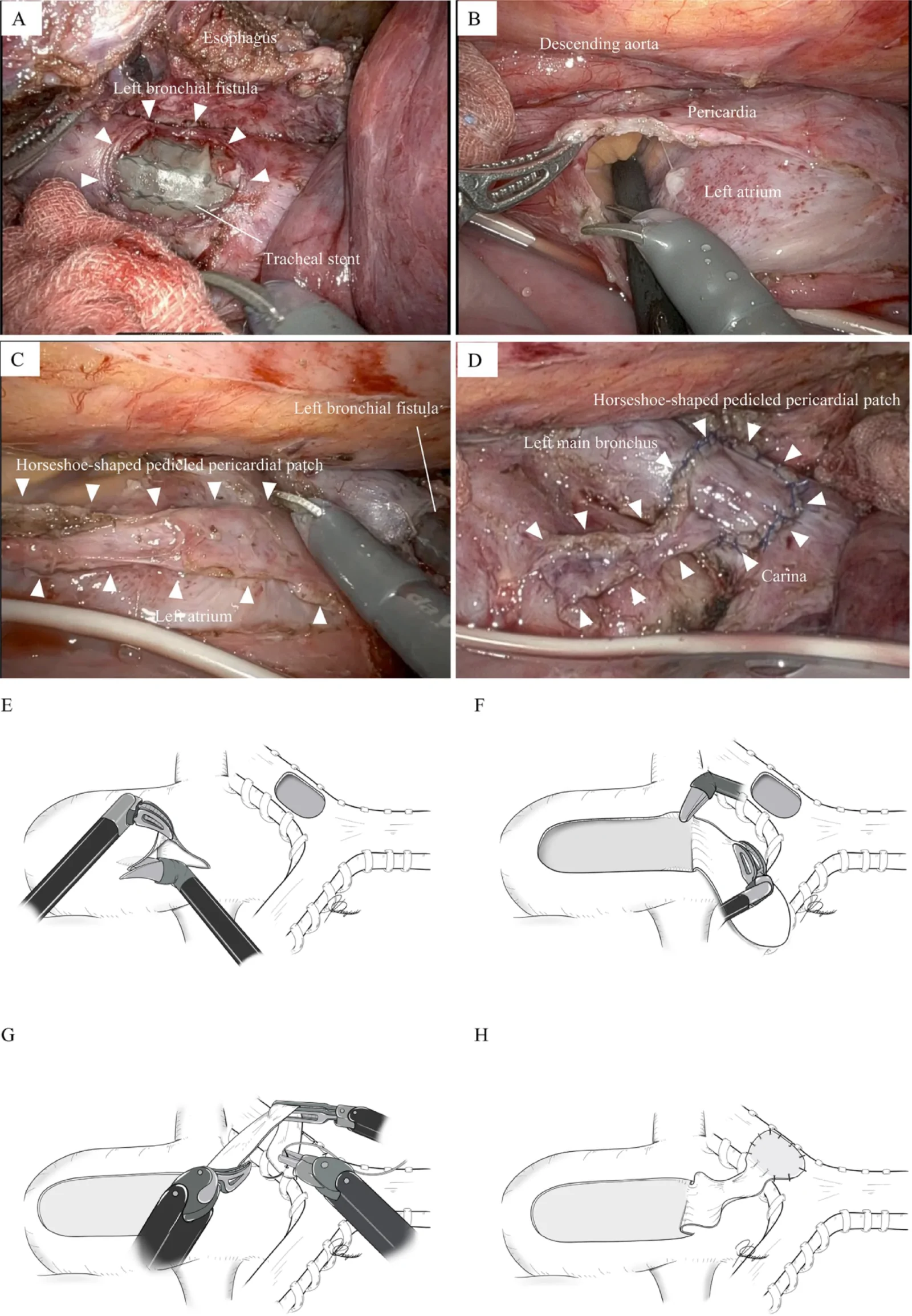

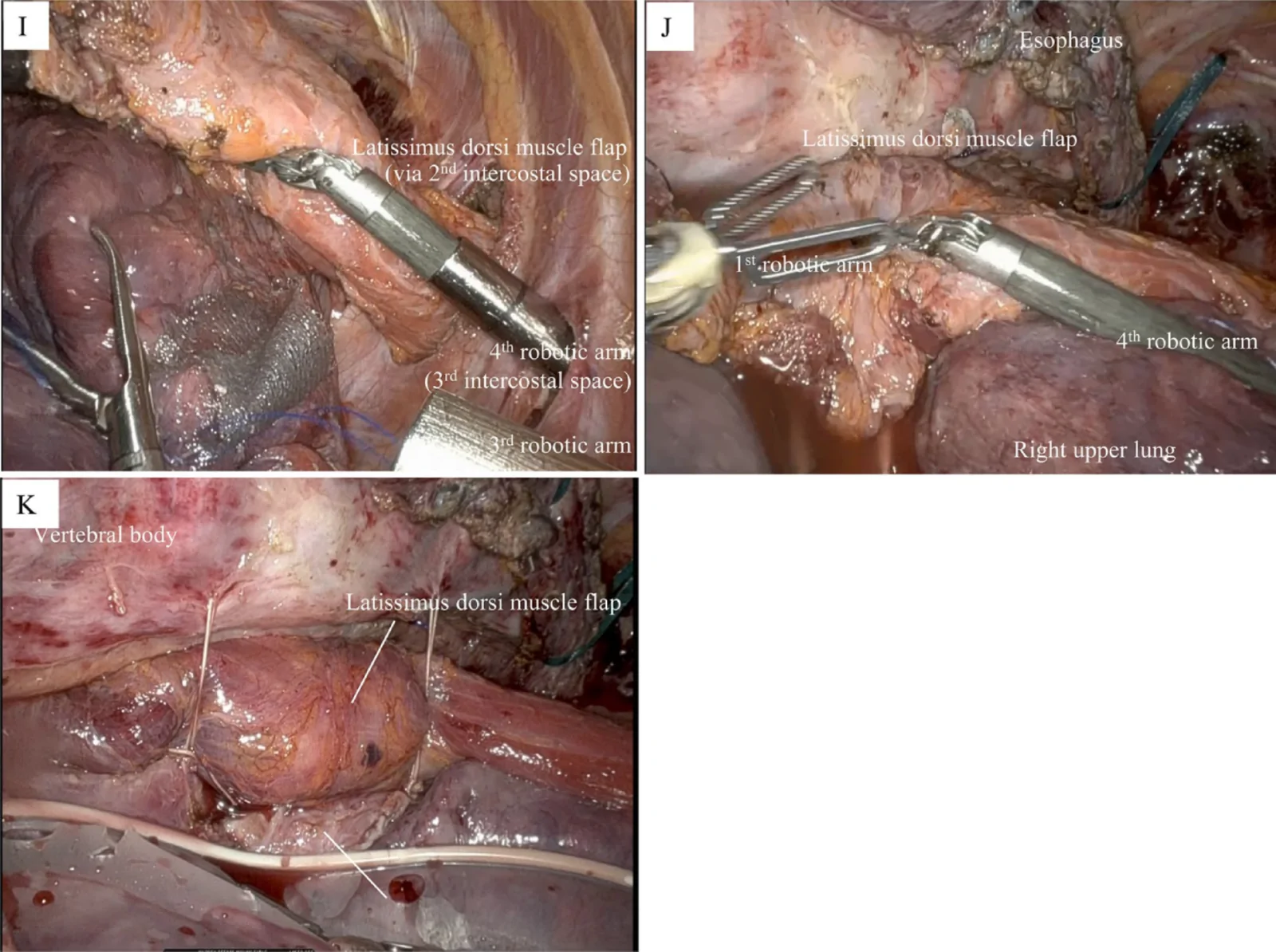
Intraoperative findings and schema of robotic fistula repair. **A** A large fistula was identified between the esophagus and the left main bronchus after mobilization of the thoracic esophagus. **B**, **C** A horseshoe-shaped pedicled autologous pericardial patch was designed near the tracheal bifurcation and harvested with sufficient length to reach the bronchial defect without tension. **D** The pedicled pericardial patch was flipped cranially and sutured to the bronchial defect. **E–H** Schematic illustration of robotic repair of the airway defect using a pedicled autologous pericardial patch. **I–K** A pedicled latissimus dorsi muscle flap was introduced into the right thoracic cavity through the second intercostal space, routed medially along the vertebral body to allow its tip to reach the bronchial fistula, and fixed over the repaired fistula to reinforce the pericardial patch

The original article has been corrected.

